# The complete chloroplast genome of *Nageia fleuryi* (Hickel) de Laub. (Podocarpaceae)

**DOI:** 10.1080/23802359.2022.2093668

**Published:** 2022-07-18

**Authors:** Xuelian Yang, Li Yan, Xia Wang, Yongfei Wu, Xiaojing Hu, Shanjun Tian

**Affiliations:** College of Agricultural, Guizhou University, Guiyang City, Guizhou Province, China

**Keywords:** *Nageia fleuryi*, complete chloroplast genome, phylogenic analysis, illumina, Podocarpaceae

## Abstract

*Nageia fleuryi* (Hickel) de Laub. 1987 belongs to the genus *Nageia* in the family Podocarpaceae and is distributed throughout southeast Asia, including China, Vietnam, and Cambodia. It is a plant with high economic beneficial for food and construction industries. Here, we report on the complete chloroplast (cp) genome of *N. fleuryi* for the first time. The complete cp genome is similar to many gymnosperm plants, however, it lacks inverted repeat regions and does not possess a typical quadripartite structure. The complete cp genome is 133,870 bp in size and the overall guanine-cytosine (GC) content was found to be 37.27%. The total number of genes is 119, including 82 protein-coding genes, 33 tRNA genes, and 4 rRNA genes. Of these, 14 genes contain one intron, two genes contain two introns, and rps12 possessed a trans-splicing mechanism. Finally, the phylogenic tree demonstrated that *N. fleuryi* is closely related to *Nageia nagi* (AB830885.1 and LC572156.1)

Belonging to genus *Nageia* in the family Podocarpaceae, *Nageia fleuryi* (Hickel) de Laub. 1987 is a plant with high economic value as a wood and oil resource (Yongbin 2013), also used as a herbal medicine to treat rheumatism and backache (Chen 2007). *N. fleuryi* is distributed throughout Southeast Asia, including China, Vietnam, and Cambodia. To date, the complete chloroplast (cp) genome of *N. fleuryi* has not been reported; therefore, in this study, we sequenced and assembled the complete cp genome of *N. fleuryi* to analyze its phylogenetic relationship for the first time.

With permission, the samples were obtained from Guizhou Botanical Garden, Guiyang, Guizhou Province, China (N 26°37′20″, E 106°43′29″) and the voucher specimen was deposited at the Laboratory of College of Agriculture in Guizhou University, Guiyang (contact: Xuelian Yang, yxl1299927812@outlook.com) under the voucher number CYZ20210701YX. Whole-genome DNA was extracted from 150 mg samples of fresh leaves following a modified CTAB protocol (Doyle 1991). The purified genomic DNA was sheared into c. 350 bp fragments to construct a paired-end (PE) library according to the Nextera XT sample preparation procedures (Illumina, San Diego, CA, USA). The PE reads of 150 bp were generated by a Novaseq 6000 sequencer (Illumina, San Diego, CA, USA). The raw data totaled 3.35 G, and the clean data totaled 3.34 G after quality control processing by NGS QC Toolkit (Patel et al. 2012), and yielded a 98-fold depth of coverage of the cp genome. The guanine-cytosine (GC) content of the clean data was 33.72%, the Q20-value was 97.78%, and the Q30 value was 93.15%, indicating a very high level of data quality for cp genome sequencing and assembly results. High-quality reads were assembled into the cp genome using the *de novo* assembler SPAdes v.3.11.0 software (Bankevich et al. 2012). Finally, the PGA program (Qu et al. 2019) was used to annotate the cp genome, using the *Nageia nagi* (GenBank accession AB830885) cp genome as the reference. We selected 24 species from NCBI to construct a maximum likelihood (ML) tree. The 77 common protein-coding genes in each complete cp genome of 24 species were aligned with the genes in *N. fleuryi* using MAFFT 7.037 (Katoh and Standley 2013) with the FFT-NS-2 strategy. Then, model-finder var 1.6 was run to select the best-fit model and the TVM++F + I + G4 model was chosen (Kalyaanamoorthy et al. 2017). Finally, RAxML var 8.2.9 was used to construct a phylogenetic tree with 1,000 bootstraps based on the ML method (Alexandros 2014). This study adhered to National Wild Plant Protective Regulations and was approved by the College of Agriculture, Guizhou Province, China.

The complete cp genome of *N. fleuryi* (GenBank accession no. OL435123) differs from Angiospermae, including Asteraceae, Solanaceae, and Rutaceae, because it lacks inverted repeat regions; thus, it is not a typical quadripartite structure. However, it was found to be similar to the complete cp genome of many gymnosperm plants, for example *Metasequoia glyptostroboides* and *Cathaya argyrophylla* (Ching-Ping et al. 2010; Chen et al. 2015)*. N. fleuryi* is 133,870 bp in size and the overall GC content was found to be 37.27%. The total number of genes was found to be 119, including 82 protein-coding genes, 33 tRNA genes, and four rRNA genes. Furthermore, 14 genes (*trnK-UUU, trnG-UCC, atpF, rpoC1, trnL-UAA, trnV-UAC, petB, petD, rpl16, rpl2, ndhB, trnI-GAU, trnA-UGC*, and *ndh*A) contain one intron, two genes (*clpP* and *ycf3*) contain two introns, and *rps*12 possess a trans-splicing mechanism. The phylogenetic relationship analysis revealed *N. fleuryi* to be closely related to *N. nagi* (AB830885.1 and LC572156.1) (Figure 1). Moreover, the genera *Afrocarpus* and *Retrophyllum* exhibited a close relationship with the genus *Nageia* compared to the other genera. This study provides useful information to aid further studies on species belonging to the genus *Nageia.*

**Figure 1.**
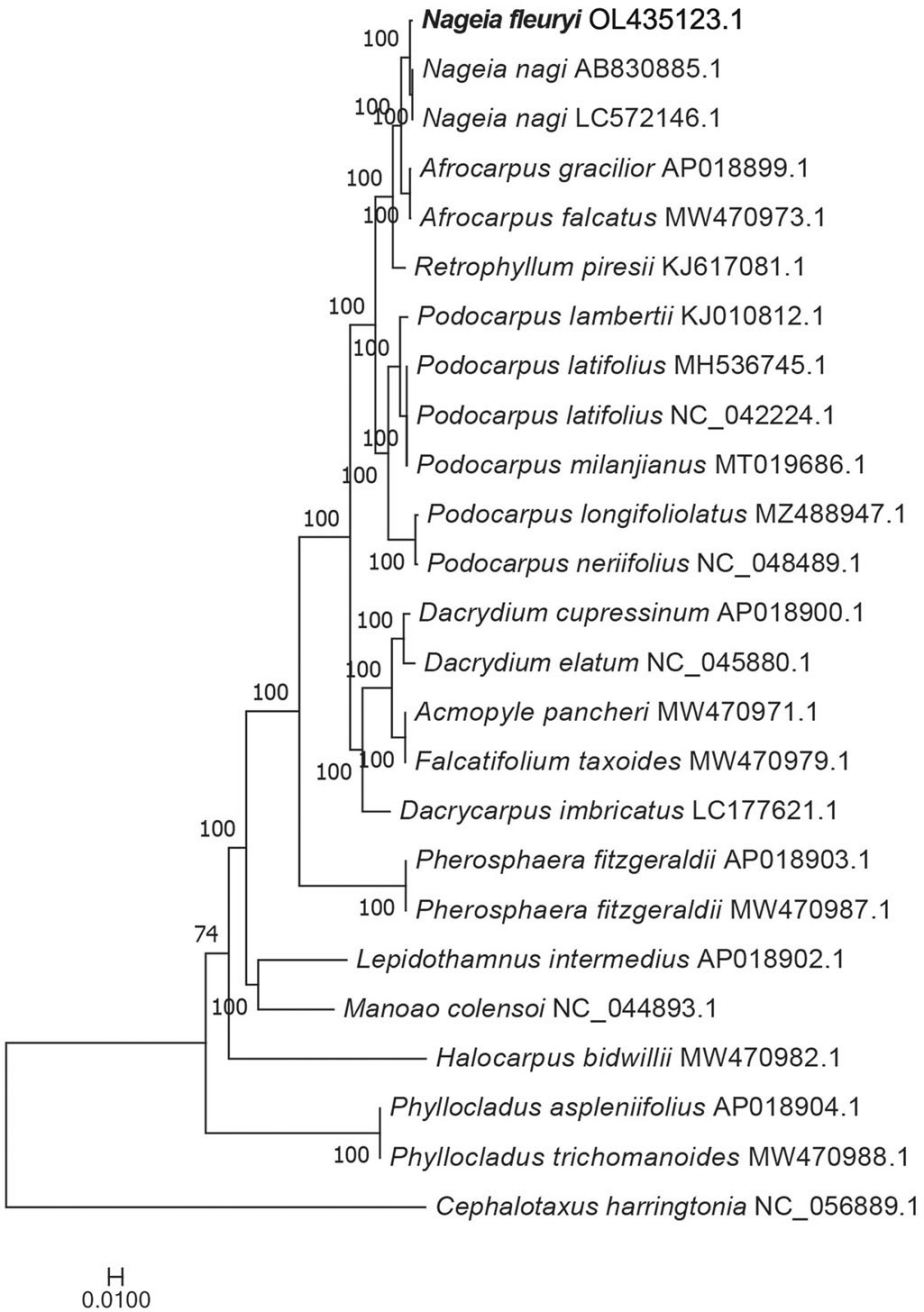
The maximum likelihood phylogenetic tree based on 77 homologous protein-coding genes in 25 species. *Note:* Numbers at the right of nodes represent the support value of 1,000 bootstrap replicates.

## Author contributions

Xue-Lian Yang and Li Yan conceived and designed the research; Xue-Lian Yang, Xia Wang, Yong-Fei Wu, Li Yan, Shanjun Tian, and Xiang-Jing Hu collected the samples, performed the experiments and analyzed the data; Shanjun Tian and Xiang-Jing Hu wrote the draft of the paper and revised the manuscript. All authors approved the final manuscript and agreed to be accountable for all aspects of the work.

## Data Availability

The genome sequence data that support the findings of this study are openly available in GenBank of NCBI at (https://www.ncbi.nlm.nih.gov/) under the accession no. OL435123. The associated BioProject, SRA, and Bio-Sample numbers are PRJNA786333, SRR17134456, and SAMN23667491, respectively.
